# A Rare Variant of Ameloblastoma Emulating as a Gingival Overgrowth: A Report of Two Cases

**DOI:** 10.7759/cureus.91298

**Published:** 2025-08-30

**Authors:** Arjun S, Roshni A, Shabina Jasmine C, Tom Thomas, Ananthu R

**Affiliations:** 1 Oral and Maxillofacial Surgery, MES Dental College and Hospital, Perinthalmanna, IND

**Keywords:** acanthomatous ameloblastoma, follicular ameloblastoma, gingival mass, odontogenic epithelium, odontogenic tumor, peripheral ameloblastoma, surgical excision

## Abstract

Peripheral ameloblastoma is a rare, benign odontogenic tumor that arises in the soft tissues of the oral mucosa. We present two cases of peripheral ameloblastoma located on the mandibular alveolar ridge, each manifesting as a painless, firm, exophytic mass initially suspected to be a reactive granulomatous lesion. Notably, the first case occurred in a patient with a prior history of intraosseous ameloblastoma in the same region, which makes this presentation clinically significant and raises important considerations regarding tumor pathogenesis and potential recurrence. Histopathological examination confirmed the diagnosis of peripheral ameloblastoma. Wide local excision was performed as the treatment of choice in both cases. The report of these cases highlights the importance of including odontogenic tumors in the differential diagnosis of persistent gingival overgrowth and highlights the need for histopathological evaluation to ensure accurate diagnosis and management.

## Introduction

Ameloblastoma is a benign odontogenic tumor arising from odontogenic epithelium, typically presenting as an intraosseous lesion in the maxilla or mandible. However, in rare instances, it can manifest in the soft tissues as an extraosseous variant known as peripheral ameloblastoma (PA) [1]. PA accounts for approximately 1-10% of all ameloblastomas, with a mean age of occurrence around 52 years [2,3]. Clinically, it is characterized by a slow-growing, painless, exophytic mass arising from the soft tissues of the tooth-bearing areas and is often misdiagnosed as a reactive gingival lesion. The most common provisional diagnoses, based on morphology, texture, and color, are epulis (42.6%) and benign tumors (26.0%), followed by papilloma and pyogenic granuloma. In denture-wearing patients, lesions on the edentulous alveolar mucosa may also be mistaken for denture irritation hyperplasia [4,5]. Although non-invasive and less aggressive than intraosseous ameloblastoma (IA), it may very rarely cause saucerization or cupping of the bone due to pressure caused by the lesion [6]. This report presents two distinct histological subtypes of PA: one exhibiting the follicular type and the other demonstrating the acanthomatous variant.

## Case presentation

Case 1

A 70-year-old female presented to the Department of Oral and Maxillofacial Surgery at MES Dental College, Kerala, with a complaint of swelling in the lower right posterior edentulous region of the jaw. She had been partially edentulous for the past 10 years and had been using a removable acrylic partial denture. The patient gave a history of experiencing discomfort from an ill-fitting denture three years ago, at which time she noticed a swelling on the lower right edentulous ridge. She was diagnosed with follicular ameloblastoma at another medical center and underwent enucleation with curettage, without any adjuvant treatment. Two months prior to the current presentation, she noticed a recurrent swelling in the same region.

**Figure 1:**
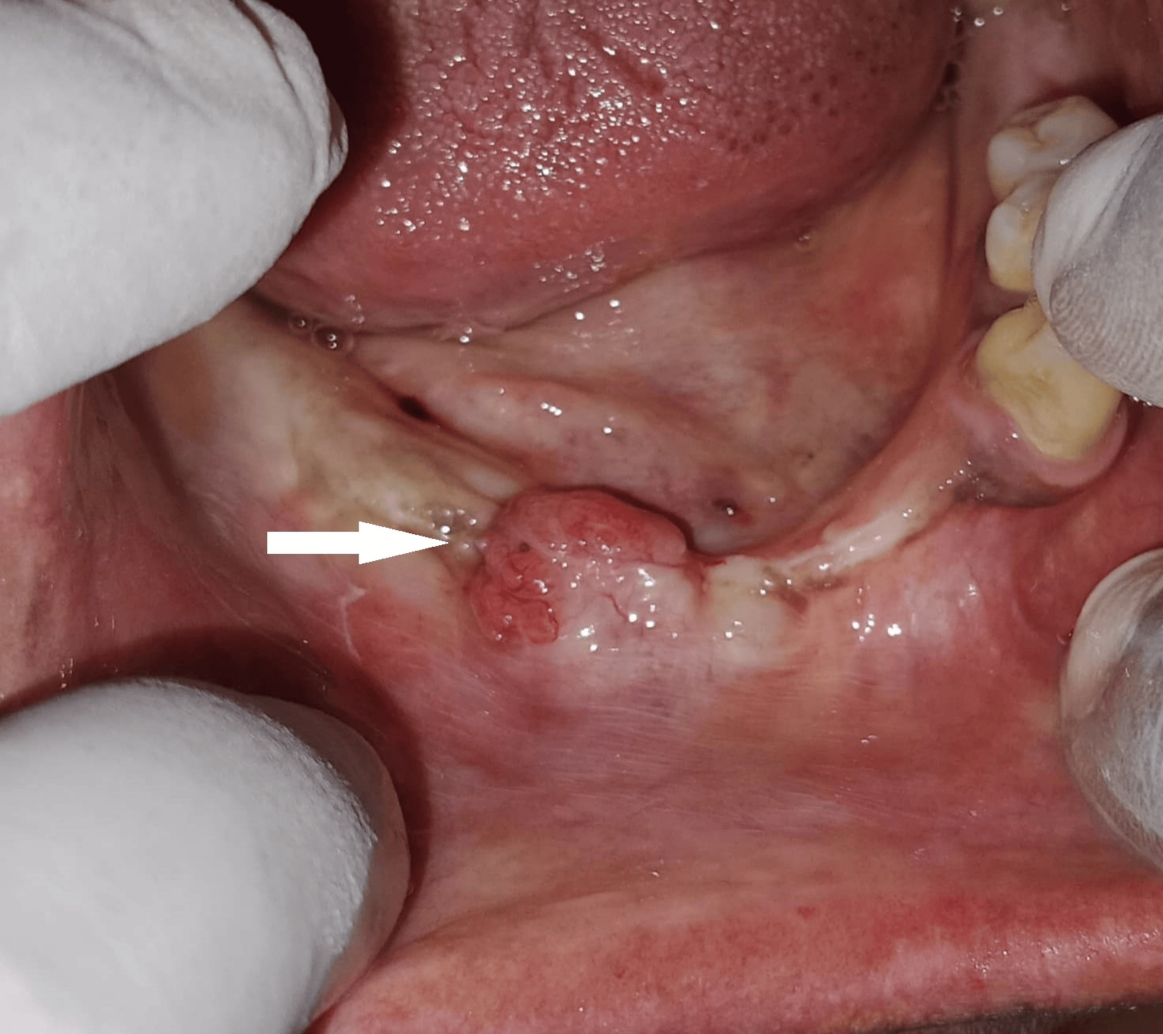
Preoperative view showing firm, sessile swelling on the right mandibular edentulous ridge.

Clinical examination revealed a pink, firm, sessile, non-tender swelling measuring approximately 1 cm × 1 cm × 1 cm on the edentulous alveolar ridge in the region of the canine and premolars on the right side. The overlying mucosa was intact, with no signs of ulceration or discharge (Figure 1).

The patient had a history of enucleation for mandibular ameloblastoma involving the right canine and premolar region three years back.

**Figure 2:**
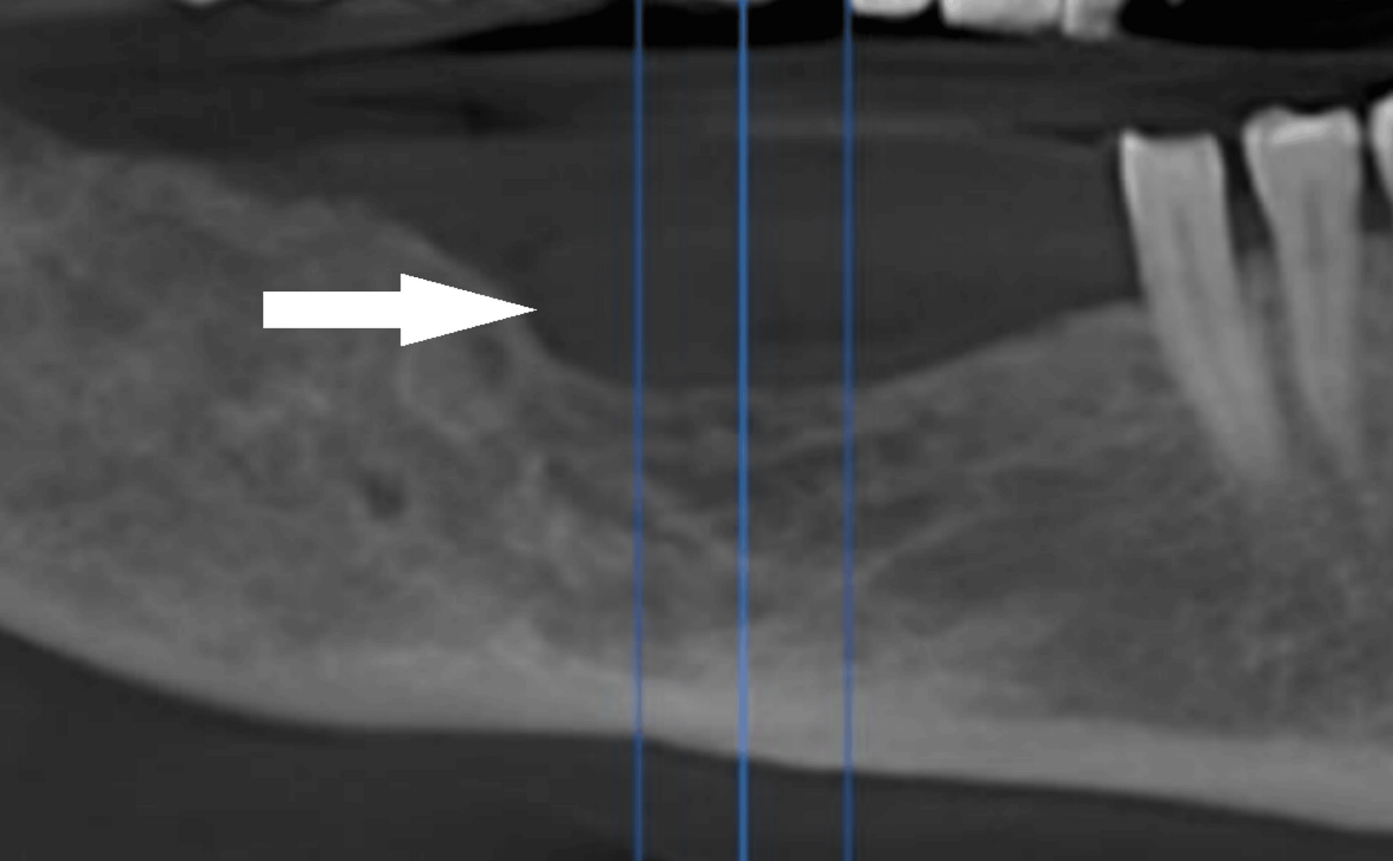
Panoramic reconstruction from CBCT showing intact crestal ridge and a surgical defect at the previous enucleation site. CBCT, cone beam computed tomography.

Cone beam computed tomography (CBCT) imaging, including panoramic reconstruction and cross-sectional views, revealed no erosive or pathological changes in the alveolar bone. A surgical defect consistent with the previous enucleation site was noted, but there was no evidence of cortical disruption or recurrence (Figure 2).

**Figure 3:**
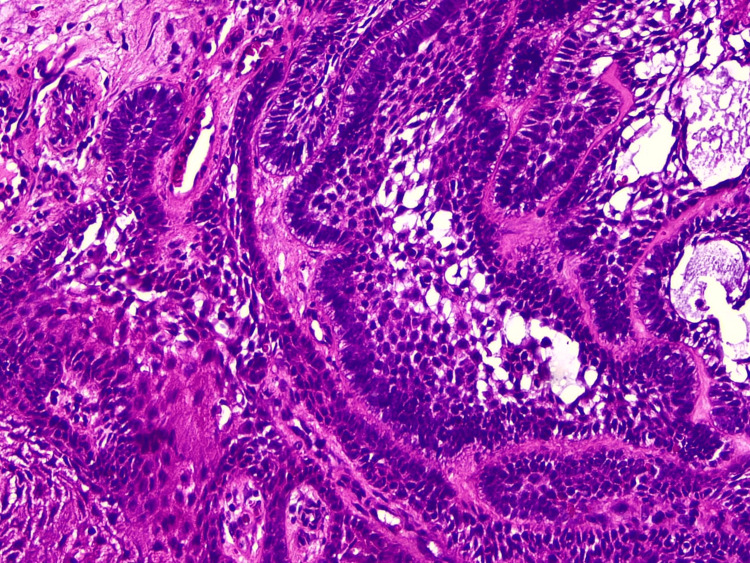
Histological section showing islands of odontogenic epithelium with peripheral palisading and central stellate reticulum-like cells beneath ulcerated squamous epithelium.

In view of the clinical findings, a wide excisional biopsy was performed under local anesthesia. Microscopic evaluation of hematoxylin and eosin (H&E, 20×)-stained sections revealed ulcerated, papillary-surfaced, keratinized, atrophic squamous epithelium overlying fibrovascular connective tissue. The underlying stroma exhibited multiple islands of odontogenic epithelium with peripheral palisaded hyperchromatic basal cells and central stellate reticulum-like cells, some showing squamous metaplasia (Figure 3).

These features were diagnostic of follicular-type PA. Regular follow-up was done at three and seven months postoperatively, with no evidence of recurrence.

Case 2

A 75-year-old male patient presented to the Department of Oral and Maxillofacial Surgery at MES Dental College, Kerala, with intermittent, dull aching pain in the lower left premolar region since one month. He reported a prior history of painless swelling in the lower left posterior teeth region since one year. The patient has a known history of asthma and uses a salbutamol inhaler as needed.

**Figure 4:**
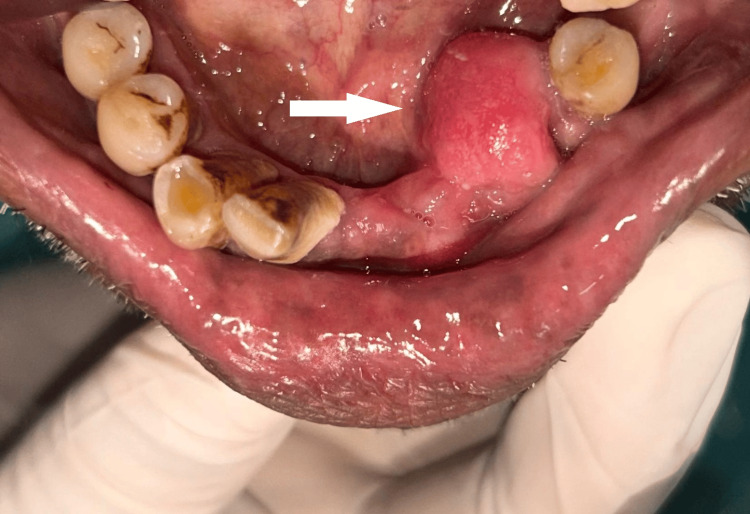
Preoperative clinical image showing a firm, sessile swelling on the lingual gingiva of the lower left canine-premolar region with intact, smooth mucosa.

Clinical examination revealed a pink, firm, sessile, non-tender swelling measuring approximately 3 cm × 3 cm × 3 cm on the lingual aspect of the lower left canine and premolars. The overlying mucosa appeared smooth, with no signs of infection, discharge, or inflammation (Figure 4).

**Figure 5:**
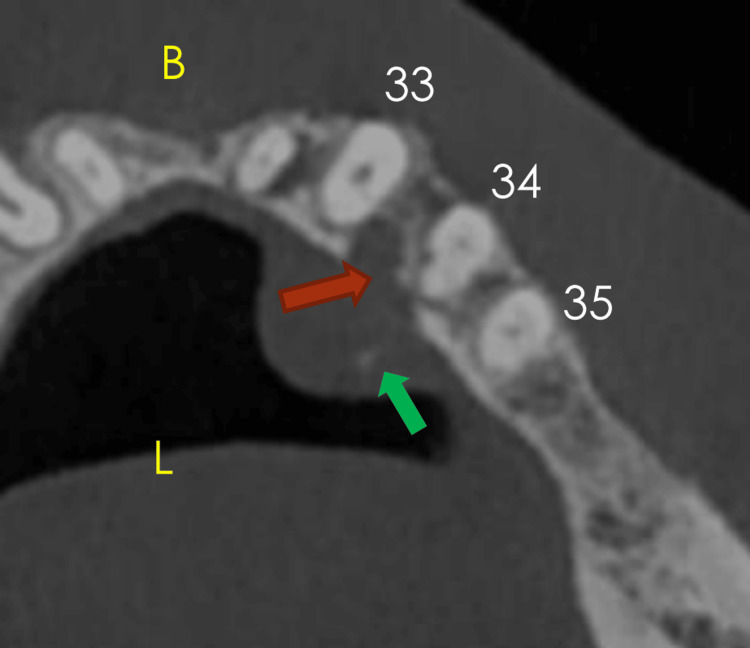
Axial CBCT image showing internal hyperdense flecks within the soft tissue lesion (green arrow) and erosion of the lingual cortical plate (red arrow). CBCT, cone beam computed tomography.

CBCT imaging demonstrated a soft tissue density lesion along the lingual alveolar region from lower left lateral incisors to second premolars, with lingual cortical erosion between the canine and first premolar. The lesion exhibited internal hyperdense flecks, which may represent remnants of destroyed lingual cortical plate, bone islands, or dystrophic calcifications (Figure 5).

An incisional biopsy was performed, and histopathological analysis confirmed the diagnosis of acanthomatous-type PA. This was followed by a wide local excision.

**Figure 6:**
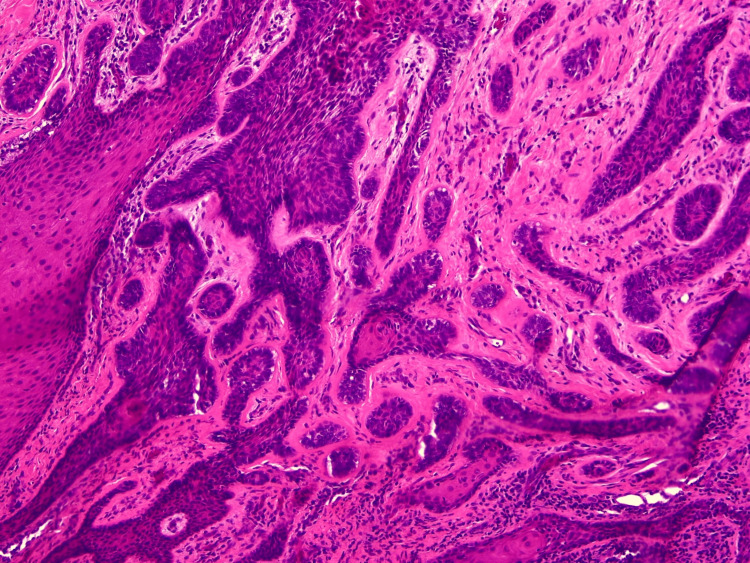
Histological section showing odontogenic epithelial islands with peripheral palisading and central squamous metaplasia.

Microscopic examination revealed keratinized stratified squamous surface epithelium overlying fibrovascular connective tissue containing multiple islands and strands of odontogenic epithelium. The islands exhibited peripheral hyperchromatic palisaded basal cells and central stellate reticulum-like cells, with most showing central squamous metaplasia. The stroma demonstrated collagen fibers with focal hyalinization (Figure 6).

**Figure 7:**
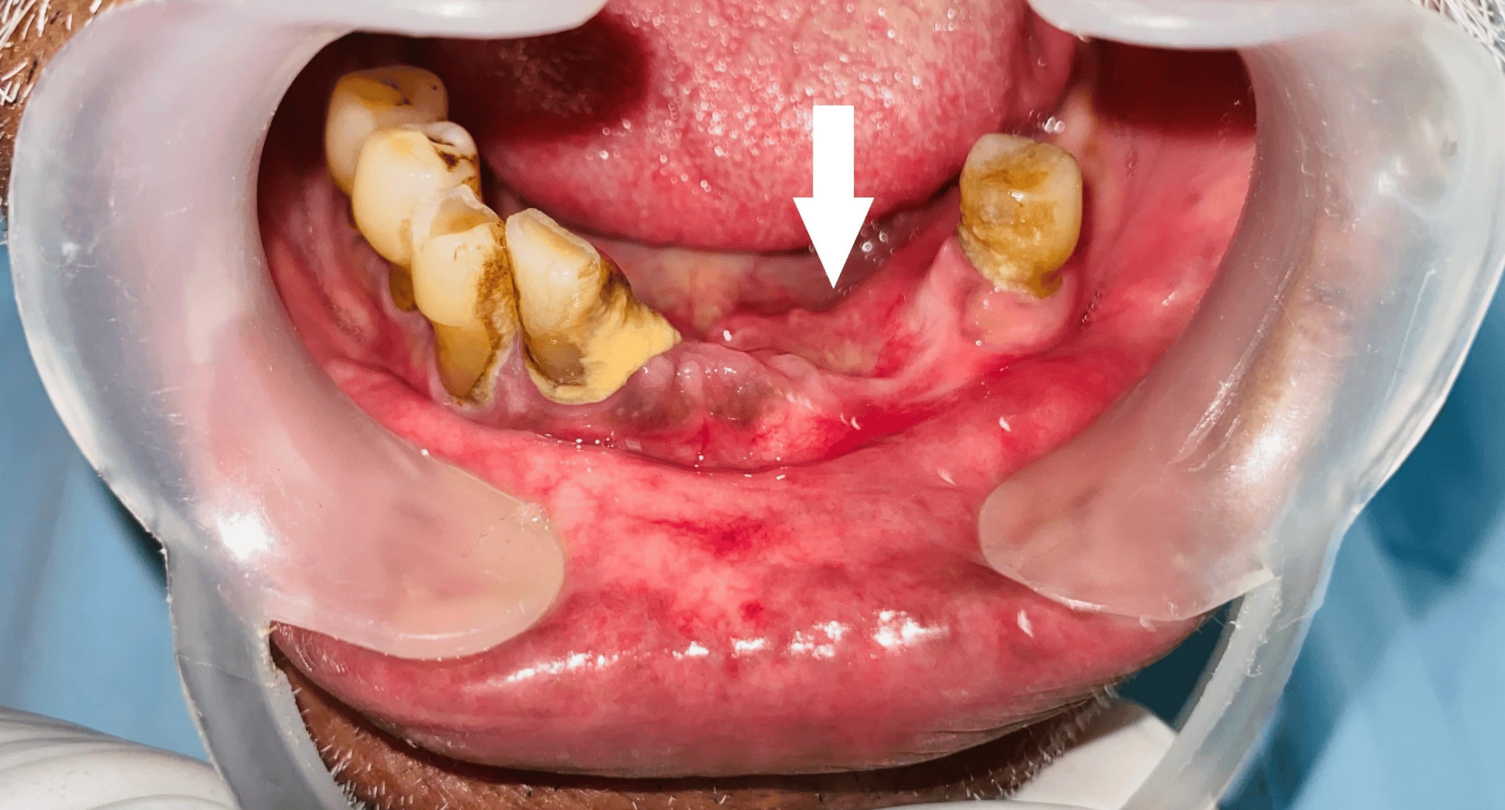
Six-month postoperative follow-up photograph showing no evidence of recurrence.

These features were consistent with acanthomatous-type PA. Follow-up was done at three and six months postoperatively, with no evidence of recurrence (Figure 7).

## Discussion

PA is a rare, benign, extraosseous variant of ameloblastoma, comprising approximately 1% to 10% of all ameloblastomas [2,3]. PA is an uncommon occurrence, and its similarity to other benign or reactive gingival lesions often delays diagnosis and complicates clinical decision-making [7].

PA most frequently affects the lingual gingiva in the mandibular premolar region and has been described in a wide age range, from 16 to 92 years. It may present as a sessile or pedunculated mass with a surface that is smooth, granular, warty, or papillary [2,8]. In our cases, the lesions appeared as a pinkish, papillary mass in the mandibular edentulous region, a classical presentation. The clinical features are nonspecific and mimic other gingival lesions such as fibrous epulis, peripheral giant cell granuloma, or pyogenic granuloma, necessitating histopathological confirmation [4].

Radiographically, PA usually does not show bone involvement. However, in a few reported cases, pressure-induced bone resorption, known as “cupping” or “saucerization,” has been documented [6]. This emphasizes the need for advanced three-dimensional imaging, such as CT or CBCT, to accurately assess lesion depth and confirm the absence of bone involvement. In our second case, CBCT revealed lingual bony erosion consistent with the characteristic cupping seen in PA.

Histopathologically, PA demonstrates islands and chords of odontogenic epithelium that resemble the central ameloblastoma, commonly with a follicular pattern showing peripheral palisading and reverse nuclear polarity [4]. Overall, PAs comprise proliferating odontogenic epithelium that can demonstrate follicular, plexiform, basal cell, or acanthomatous patterns, with follicular and acanthomatous being the most frequently observed configurations [5]. Although acanthomatous changes are generally less common, they were predominant in our second case.

PA arises from the remnants of the dental lamina (glands of Serres), basal cells of the oral epithelium, or minor salivary gland ductal cells. While most lesions are found on the gingiva, reports of extragingival sites such as the buccal mucosa, floor of the mouth, tongue, and even infratemporal fossa support a pluripotent origin [4]. Rare reports of PA in the temporal fossa highlight its potential to present in atypical locations, further complicating clinical diagnosis [9,10].

The recurrence rate of PA ranges from 9% to 20%, which is lower than that of conventional IAs; however, incomplete excision may contribute to recurrence [8,9]. In the first case, the patient had a prior history of IA treated with enucleation and curettage at another hospital. Literature demonstrates that patients treated with enucleation alone are more prone to recurrence, whereas complete excision with adequate margins significantly reduces this risk [11,12].

In Case 1, a key diagnostic challenge lies in distinguishing between a true de novo PA and a soft tissue recurrence of the patient’s previously treated IA. Distinguishing between these entities is clinically significant, as IAs carry a markedly higher recurrence risk and require prolonged surveillance. While PA generally arises independently in the soft tissues of the gingiva, the possibility of recurrence in this case cannot be excluded, particularly given the lesion’s occurrence in the same anatomical region.

The preferred treatment for PA is surgical excision with adequate tumor-free margins. Although extensive resection is not typically indicated, thorough histological margin evaluation is critical to minimize the risk of recurrence [4,6,8,13]. In both cases, complete surgical excision was performed with adequate margins, and the patients have been under regular follow-up with no signs of recurrence. Late recurrences have been documented, and although extremely rare, malignant transformation into ameloblastic carcinoma or metastasizing ameloblastoma has been reported in less than 1% cases, reinforcing the need for long-term follow-up [2,4,8].

Given the rarity of PA and the subtle distinctions between cases, a structured comparison is useful to highlight similarities and differences in presentation, diagnostic approach, and management (Table 1).

**Table 1:** Comparative summary of demographic, clinical, imaging, histopathological, and treatment characteristics of the two reported cases of peripheral ameloblastoma. CBCT, cone beam computed tomography.

Parameter	Case 1	Case 2
Age/Sex	70-year-old female	75-year-old male
Relevant History	Prior intraosseous follicular ameloblastoma in the same region, treated with enucleation and curettage at another hospital, no adjuvant therapy	History of painless swelling in the lower left posterior region for one year
Chief Complaint	Swelling in the lower right posterior edentulous ridge, noticed two months prior	Intermittent dull aching pain in the lower left premolar region for one month
Clinical Examination	Firm, sessile, non-tender swelling (1 × 1 × 1 cm) on edentulous ridge; intact mucosa	Firm, sessile, non-tender swelling (3 × 3 × 3 cm) on lingual aspect of lower left canine-premolars; smooth mucosa, no discharge
Imaging	CBCT cross-sectional and panoramic reconstruction; no erosive or pathological bony changes	CBCT axial and panoramic reconstruction; soft tissue density lesion with internal hyperdense flecks, lingual cortical erosion
Histopathology	Follicular-type peripheral ameloblastoma; islands of odontogenic epithelium with peripheral palisaded basal cells and central stellate reticulum-like cells	Acanthomatous-type peripheral ameloblastoma; odontogenic islands with peripheral palisading, central squamous metaplasia, stromal hyalinization
Treatment	Wide local excision with adequate margins	Incisional biopsy followed by wide local excision with adequate margins
Follow-up	Regular follow-up, no recurrence to date	Regular follow-up, no recurrence to date

At the molecular level, mutations in the mitogen-activated protein kinase (MAPK) pathway, including BRAF V600E, FGFR2, and NRAS, have been identified in ameloblastomas, including PA [4]. Among these, the BRAF V600E mutation is the most prevalent, reported in approximately 62% of ameloblastomas, with a higher frequency in mandibular lesions [14]. Molecular profiling is increasingly valuable in understanding the biological behavior of ameloblastomas and in guiding potential targeted therapies.

A key limitation in the present report, particularly concerning Case 1, is that molecular comparison between the previously treated IA and the current soft tissue lesion was not feasible in our setting. This constraint limited definitive classification of the lesion as either a true de novo PA or a soft tissue recurrence.

Advances in molecular biology have highlighted recurrent somatic and activating mutations, particularly within the MAPK and sonic hedgehog (SHH) signaling pathways, paving the way for targeted therapeutic approaches [14]. Current clinical applications of targeted therapy for ameloblastoma primarily focus on BRAF V600E, with most evidence derived from case reports and small case series [15]. Agents that have been utilized include the BRAF inhibitors dabrafenib and vemurafenib, the MEK inhibitor trametinib, and combination regimens such as dabrafenib with trametinib. Although still investigational, targeted therapy represents a promising option, either in the neoadjuvant setting to reduce surgical morbidity or as a primary treatment for metastatic, unresectable, or multiply recurrent ameloblastomas [14].

## Conclusions

PA is a rare but clinically significant odontogenic tumor that necessitates careful distinction from reactive gingival lesions, other odontogenic neoplasms, and, in patients with a prior history of IA, the possibility of soft tissue recurrence. This diagnostic complexity highlights the value of integrating advanced imaging modalities such as CBCT and MRI to detect subtle bone involvement and guide surgical planning. Histopathological examination remains the gold standard for definitive diagnosis, often supplemented by immunohistochemistry to exclude morphologically similar lesions. Conservative surgical excision with adequate margins is the treatment of choice, as it typically provides excellent outcomes while preserving surrounding structures. However, long-term follow-up is essential due to the potential for late recurrences and the rare possibility of malignant transformation into ameloblastic carcinoma or metastasizing ameloblastoma. Early recognition, precise histopathological diagnosis, and careful long-term postoperative monitoring are critical to ensuring optimal patient outcomes in cases of PA.

## References

[1] Gardner DG (1996). Some current concepts on the pathology of ameloblastomas. Oral Surg Oral Med Oral Pathol Oral Radiol Endod.

[2] Ülker E, Kirtiloğlu T, Taban B (2020). Peripheral ameloblastoma: A case report. J Clin Exp Dent.

[3] Nosé V, Lazar AJ (2022). Update from the 5th Edition of the World Health Organization Classification of Head and Neck Tumors: Familial tumor syndromes. Head Neck Pathol.

[4] Decani S, Quatrale M, Caria V, Moneghini L, Varoni EM (2024). Peripheral ameloblastoma: A case report and review of literature. J Clin Med.

[5] Philipsen HP, Reichart PA, Nikai H, Takata T, Kudo Y (2001). Peripheral ameloblastoma: Biological profile based on 160 cases from the literature. Oral Oncol.

[6] Zhang X, Tian X, Hu Y, Zhang C, Wei C, Yang X (2018). Oral peripheral ameloblastoma: A retrospective series study of 25 cases. Med Oral Patol Oral Cir Bucal.

[7] Janardhanan M, Rakesh S, Savithri V, Aravind T (2018). Peripheral ameloblastoma with neoplastic osseous invasion versus peripheral intraosseous ameloblastoma: A challenging diagnosis. J Oral Maxillofac Pathol.

[8] Anpalagan A, Tzortzis A, Twigg J, Wotherspoon R, Chengot P, Kanatas A (2021). Current practice in the management of peripheral ameloblastoma: A structured review. Br J Oral Maxillofac Surg.

[9] Hashemi H, Näsman A, Farzad P (2022). Peripheral ameloblastoma presenting as a solid mass in the temporal fossa: A case report and review of the literature. Oral Maxillofac Surg Cases.

[10] Sansgiri TS, Saluja H, Shah S, Dadhich A, Patil R (2024). Recurrent ameloblastoma presenting as a solid mass in temporal fossa: A unique case report with diverse histological patterns. J Maxillofac Oral Surg.

[11] Olaitan AA, Arole G, Adekeye EO (1998). Recurrent ameloblastoma of the jaws. A follow-up study. Int J Oral Maxillofac Surg.

[12] Hertog D, Schulten EA, Leemans CR, Winters HA, Van der Waal I (2011). Management of recurrent ameloblastoma of the jaws; a 40-year single institution experience. Oral Oncol.

[13] Ghai S (2022). Ameloblastoma: An updated narrative review of an enigmatic tumor. Cureus.

[14] Graillon N, Akintoye SO, Iocca O, Kaleem A, Hajjar S, Imanguli M, Shanti RM (2023). Current concepts in targeted therapies for benign tumors of the jaw - A review of the literature. J Craniomaxillofac Surg.

[15] Yoithapprabhunath TR, Srichinthu KK, Gupta D, Singh D, Pasupuleti S, Nirmal RM (2022). Effectiveness of molecular-targeted chemotherapy in ameloblastomas: A systematic review. Indian J Dent Res.

